# Multivariable Risk Modelling and Survival Analysis with Machine Learning in SARS-CoV-2 Infection

**DOI:** 10.3390/jcm12227164

**Published:** 2023-11-18

**Authors:** Andrea Ciarmiello, Francesca Tutino, Elisabetta Giovannini, Amalia Milano, Matteo Barattini, Nikola Yosifov, Debora Calvi, Maurizo Setti, Massimiliano Sivori, Cinzia Sani, Andrea Bastreri, Raffaele Staffiere, Teseo Stefanini, Stefania Artioli, Giampiero Giovacchini

**Affiliations:** 1 Nuclear Medicine Unit, Ospedale Civile Sant’Andrea, Via Vittorio Veneto 170, 19124 La Spezia, Italy; francesca.tutino@asl5.liguria.it (F.T.); elisabetta.giovannini@asl5.liguria.it (E.G.); nikola.yosifov@asl5.liguria.it (N.Y.); giampiero.giovacchini@asl5.liguria.it (G.G.); 2 Oncology Unit, Ospedale Civile Sant’Andrea, 19124 La Spezia, Italy; amalia.milano@asl5.liguria.it; 3 Radiology Unit, Ospedale Civile Sant’Andrea, 19124 La Spezia, Italy; matteo.barattini@asl5.liguria.it (M.B.); teseo.stefanini@asl5.liguria.it (T.S.); 4 Infectius Diseases Unit, Ospedale Civile Sant’Andrea, 19124 La Spezia, Italy; debora.calvi@asl5.liguria.it (D.C.); stefania.artioli@asl5.liguria.it (S.A.); 5 Internal Medicine Unit, Ospedale San Bartolomeo, 19138 Sarzana, Italy; maurizio.setti@asl5.liguria.it; 6 Pneumology Unit, Ospedale Civile Sant’Andrea, 19124 La Spezia, Italy; massimiliano.sivori@asl5.liguria.it; 7 Intensive Care Unit, Ospedale Civile Sant’Andrea, 19124 La Spezia, Italy; cinzia.sani@asl5.liguria.it; 8 Emergency Department, Ospedale Civile Sant’Andrea, 19124 La Spezia, Italy; andrea.bastreri@asl5.liguria.it; 9 Emergency Department, Ospedale San Bartolomeo, 19138 Sarzana, Italy; raffaele.staffiere@asl5.liguria.it

**Keywords:** SARS-CoV-2, machine learning, radiomics, CT, survival

## Abstract

Aim: To evaluate the performance of a machine learning model based on demographic variables, blood tests, pre-existing comorbidities, and computed tomography(CT)-based radiomic features to predict critical outcome in patients with acute respiratory syndrome coronavirus 2 (SARS-CoV-2). Methods: We retrospectively enrolled 694 SARS-CoV-2-positive patients. Clinical and demographic data were extracted from clinical records. Radiomic data were extracted from CT. Patients were randomized to the training (80%, n = 556) or test (20%, n = 138) dataset. The training set was used to define the association between severity of disease and comorbidities, laboratory tests, demographic, and CT-based radiomic variables, and to implement a risk-prediction model. The model was evaluated using the C statistic and Brier scores. The test set was used to assess model prediction performance. Results: Patients who died (n = 157) were predominantly male (66%) over the age of 50 with median (range) C-reactive protein (CRP) = 5 [1, 37] mg/dL, lactate dehydrogenase (LDH) = 494 [141, 3631] U/I, and D-dimer = 6.006 [168, 152.015] ng/mL. Surviving patients (n = 537) had median (range) CRP = 3 [0, 27] mg/dL, LDH = 484 [78, 3.745] U/I, and D-dimer = 1.133 [96, 55.660] ng/mL. The strongest risk factors were D-dimer, age, and cardiovascular disease. The model implemented using the variables identified using the LASSO Cox regression analysis classified 90% of non-survivors as high-risk individuals in the testing dataset. In this sample, the estimated median survival in the high-risk group was 9 days (95% CI; 9–37), while the low-risk group did not reach the median survival of 50% (*p* < 0.001). Conclusions: A machine learning model based on combined data available on the first days of hospitalization (demographics, CT-radiomics, comorbidities, and blood biomarkers), can identify SARS-CoV-2 patients at risk of serious illness and death.

## 1. Introduction

Severe acute respiratory syndrome coronavirus disease 2 (SARS-CoV-2) has had a significant economic and global health impact and continues to be a major concern as new variants are identified [1,2,3]. Moreover, global environmental changes could increase the probability of future pandemics, necessitating the development and application of accurate and innovative tools for risk stratification [4].

The clinical disease phenotype for SARS-CoV-2 is extremely heterogeneous. The infection can proceed asymptomatically or evolve with differing intensities up to the severe disease that is associated with a low survival rate [5,6].

The variability of clinical manifestations makes outcome prediction particularly difficult. This can be a major issue when the volume of patients is high and resources are limited, as occurs during a pandemic. Therefore, the identification of major risk factors and the implementation of an outcome-prediction model could support treatment planning and optimal resource allocation.

To date, several studies have reported on the association between the mortality rate and the subject’s age, pre-existing comorbidities, some blood biomarkers, and the degree of lung involvement (mainly based on computed tomography (CT) scans) [5,7,8].

The data published so far have highlighted greater frailty in older adults, who seem to have a higher rate of severe disease and mortality than young patients [9]. 

There is broad agreement in the literature that comorbidities are present in approximately half of patients with SARS-CoV-2. According to Richardson et al., coronary heart disease, hypertension, diabetes, and chronic obstructive lung disease are significantly associated with increased mortality [5,10].

Several blood biomarkers have been associated with SARS-CoV-2. High D-dimer levels have been reported as predictors of mortality in hospitalized patients [11]. Similarly, some blood biomarkers of inflammation, such as C-reactive protein (CRP), and cell damage, such as lactate dehydrogenase (LDH), appear to be significantly increased in the most severe forms of the disease [12].

The degree of lung involvement, as primarily assessed using CT, is a potential predictor of outcome [8,13,14,15,16,17]. Data of potential clinical interest contained in the medical images can be read by expert radiologists or extracted using dedicated software; the latter approach is known as radiomics. Recently, several studies have proposed that radiomics and deep learning methods can be used to distinguish normal lung parenchyma from that affected by SARS-CoV-2 pneumonia [18] or to predict patient diagnosis [19] and outcome [20]. These approaches offer high diagnostic performance, as evidenced by the area under the receiver-operating characteristic curve (AUC ≥ 89%) [20,21].

Radiomics data were extracted with Pyradiomics, which is an open-source software implemented in Python 3.6 able to extract radiomics features from two- or three-dimensional medical images [22]. This platform has been widely used by several researchers to evaluate the predictive value of radiomics in several diseases including SARS-CoV-2 pneumonia [23,24,25].

The current study aims to implement and validate a mortality-risk-prediction model for SARS-CoV-2 based on demographic data, blood biomarkers, baseline comorbidities, and radiomic CT data using machine learning methods.

## 2. Materials and Methods

### 2.1. Population

This retrospective study was based on clinical records from patients admitted to hospital services through the emergency department. These patients exhibited fever, sore throat, dry cough, diarrhoea, loss of taste or smell, chest pain, and/or shortness of breath or breathing difficulty between 1 March 2020 and 31 December 2020. The regional review committee granted ethical approval (CER Liguria: 553/2020/10988) for this study, and written informed consent was waived. We deidentified data to avoid any potential breach of patient privacy and processed it for research purposes from 1 April 2021 to 31 December 2021. We retrieved CT images from the hospital’s picture-archiving and communications systems (PACs). Inclusion criteria included (i) positive RT-PCR assay for COVID-19 and (ii) at least one non-contrast chest CT. For patients with multiple RT-PCR tests or CT scans, we used the test closest to the time of initial presentation to the emergency department. Exclusion criteria included (i) patients without basal CT, (ii) diagnosis of pneumonia with SARS-CoV-2 not confirmed, and (iii) CT images deteriorated by motion artifact. The study cohort consisted of 694 subjects with RT-PCR confirmed diagnosis of COVID-19 pneumonia, encompassing 447 males and 247 females.

Overall survival (OS) was defined as the time from the first hospital presentation to the date of death or censoring. Patients who were alive were censored at their last follow-up to 31 December 2020. We used hospital records to determine the status of the patients. 

### 2.2. CT-Acquisition Parameters and Interpretation

All patients underwent non-enhanced chest CT. Images were acquired in supine position on Aquilion (Toshiba Medical Systems, Tokyo, Japan) and Optima CT660 (GE Healthcare, Milwaukee, WI, USA) multi-detector CT scanners (120 kVp; 120–440 mAs; thickness: 5–7 mm; slice interval: 5 m; rotation speed: 0.5–1.0 s; helical pitch 1.0875:1 or 1.375:1). Images were reconstructed at 512 × 512 pixels, with a section width of 0.625 mm. CT images were reviewed in S. Andrea Hospital’s imaging laboratory by two board-certified radiologists with approximately 5 years of experience in chest-CT reading. CT images were classified according to the criteria proposed by the Radiological Society of North America (RSNA) into two classes: “typical” and “atypical” findings, as defined by Simpson and colleagues [26].

### 2.3. Image Analysis and Texture Features Extraction

Lung images were segmented using the 3D slicer software v4.11 [27]. Two certified radiologists reviewed all segmented images to rule out segmentation errors. 

In compliance with the Imaging Biomarker Standardisation Initiative (IBSI) protocols (https://arxiv.org/abs/1612.07003 accessed on 24 July 2023), we applied intensity discretisation and spatial resampling before feature extraction. Images were discretised with a 64-bin width and resampled to 2 × 2 × 2 mm^3^ voxel size with B-spline interpolation. Before analysis, we applied the ComBat harmonisation method [28] to extracted features to remove batch effects from different scanners’ images, using the “neuroComBat 1.0.13” package in R. We applied ComBat harmonisation to the training dataset alone, and subsequently applied estimates obtained from training data harmonisation to the test set. Radiomic features were extracted in the open-source software package Pyradiomics (https://github.com/Radiomics/pyradiomics/releases, accessed on 9 October 2023 v3.1.0), and a total of 43 features were extracted from each CT lung image [22]. Among them were three first-order statistical features, nine Gray-level co-occurrence matrices (GLCM), thirteen Gray-level run-length matrices (GLRLM), thirteen Gray-level size-zone matrices, a Gray difference matrix (GLSZM), and five Gray difference matrix features (NGTDM).

### 2.4. Feature Selection and Classification

Least absolute shrinkage and selection operator (LASSO) regularized Cox regression [29] was used to build the model for predicting overall patient survival with the demographic, laboratory, and radiomic features [30]. The LASSO method is used for its firm ability to reduce the number of predictors by selecting only those with higher predictive performance [31]. Moreover, LASSO regression is reported among the most commonly used techniques for radiomic-feature selection [32].

LASSO regularized Cox regression was used to choose the most relevant predictors under the Cox proportional-hazard model, using a penalty term for the estimation of the partial maximum likelihood. This penalty reduces the coefficient values forcing them close or equal to zero for those affecting the model least. The penalty can be tuned using a constant called lambda (λ). The best lambda, was defined as the lambda that minimize the 10-fold cross-validation prediction error. We performed regularised Cox regression with cv.glmnet under the R 4.1.3 (http://www.r-project.org, accessed on 9 October 2023) glmnet package 4.1-4 [33].

The cv.glmnet function provides the cross-validated mean C-index and C-index standard error estimate. The function also reports the minimum mean cross-validated error (lambda.min) and the value of lambda, providing the most regularised model, with a cross-validated error within 1 standard error of the minimum [30]. C-statistic was used to assess the predictive performance of the LASSO–Cox regression model.

### 2.5. Model Design

The LASSO’s initial selection included 57 predictors. Among these were two demographic factors (age and gender), three laboratory tests (C-Reactive Protein, Lactate Dehydrogenase, and D-dimer), nine comorbidities (cancer, blood cancer, diabetes, obesity, haematological disease, cardiovascular disease, cerebrovascular disease, and chronic obstructive pulmonary disease), and forty-three radiomic features. We implemented the predictive model with the demographic, metabolic, and radiomic characteristics that survived the LASSO analysis with the Cox multiple regression method.

**Figure 1:**
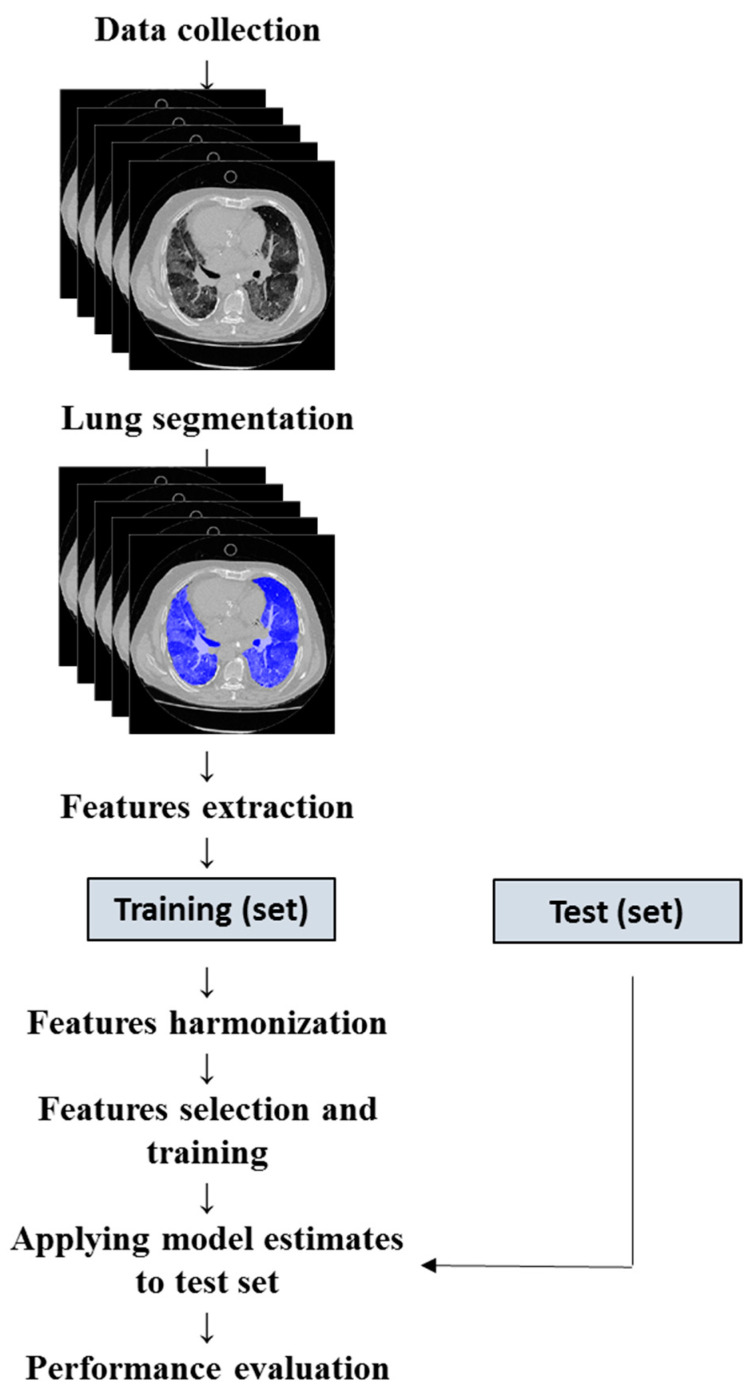
Flowchart for machine learning model development.

To evaluate the model’s performance on new data not used for training, we divided the cohort randomly to include 80% of the sample in the training set and 20% in the validation set [34]. We subsequently evaluated the performance by applying the estimated training parameters to the testing data (Figure 1).

### 2.6. Model Validation and Calibration

Regression Modeling Strategies (RMS v6.6) is an open-source package implemented under R, containing a collection of functions aimed at evaluating the performance of predictive models [35]. The validation and calibration of the model obtained from the LASSO–Cox regression was carried out with the “validated” and “calibrated” functions included in the RMS package. We used the calibration method to evaluate the performance of the prediction model by comparing the predicted with the observed probabilities. To reduce overfitting and quantify optimism, we internally validated the model by computing an optimism-corrected C-statistic after 1000 bootstrapped resampling. Validation was performed using a test dataset. Model calibration and validation were based on C-index and Brier score metrics. After validation, we calculated each patient’s individual risk score using the ggrisk v1.3 package. Subjects were placed in high- and low-risk groups based on the median risk score. 

To evaluate the ability of the risk score to stratify patients into clinically relevant classes, we used Kaplan–Meier to estimate the fraction of subjects who survived in high- and low-risk groups. 

### 2.7. Statistical Analysis

We used R software (version 4.1.3, http://www.r-project.org, accessed on 9 October 2023) for data analysis and graphics. We tested continuous data using independent t-tests, with degrees of freedom adjusted for inequality of variance where appropriate. Wald’s test was used to evaluate the relative importance of each predictor with the outcome. This measure ranges from 0 to ∞ as the association of the predictors with outcome increases, allowing comparison of continuous and categorical variables [35,36].

We conducted LASSO logistic regression analysis using the glmnet package in R. The survival curves were generated using the Kaplan–Meier method implemented in the ggsurvplot function. Validation plots were produced using the root mean squares (RMS) package. 

We used the c-statistic to evaluate the discrimination ability of the model. The c-statistic is defined as the proportion of subjects in whom the rankings of predicted and observed survival times agree [35]. C-statistics of > 0.80, between 0.70 and 0.80 and ≤0.50 indicate good, acceptable, and low model discrimination ability, respectively.

The Brier score is defined as the average squared difference between actual events and predicted probabilities. The values range from 0 to 1, with the extremes of 0 and 1 indicating perfect or totally inaccurate agreement between predicted and observed events [37].

We used chi-square analysis for categorical variables. We calculated the 95% confidence intervals (CIs) for sensitivity (SS), specificity (SP), odds ratio (OR), positive predictive value (PPV), and negative predictive value (NPV) to estimate how strongly the model-predicted diagnosis was associated with clinical outcome. Two-tailed *p* values of less than 0.05 were considered statistically significant. 

## 3. Results

We recruited a total of 694 patients and randomized them to include 80% (n = 556) in training and 20% (n = 138) in test datasets. Table 1 summarizes the patient characteristics. The median age was 64 years (age range: 20–107 years). The study sample consisted predominantly of males (64%). Most patients were residents of northeastern Italy. The median hospital stay was 11 days (range: 3–86 days). Patients had a median CRP of 3.00 mg/dL (range: 0.11–37.00 mg/dL) and a median LDH of 486 U/I (range: 78–3745 U/I). Patients had a median D-dimer of 1133 ng/mL (range: 96–152,015 ng/mL). Deceased patients were predominantly male (66%) and more than 50 years old. Compared with survivors, deceased patients showed differences in laboratory findings (Table 1). As expected, in the current study sample, D-dimer, CRP and LDH were also significantly increased in non-survivors compared with survivors (Table 1). Visual assessment of CT images according to RSNA guidelines [26] identified 111 of 157 non-survivors and 299 of 537 survivors had typical findings. Table 2 shows the impact of pre-existing comorbidities on mortality in patients with SARS-CoV-2. Cardiovascular and cerebrovascular diseases, cancer, haematological diseases and chronic obstructive pulmonary disease all significantly increased the probability of death in the study sample.

Figure S1 (see Supplemental Material) shows the relevance of each predictor based on the Wald test. This relevance was obtained from the multivariable logistic regression used for modelling patient mortality. Predictors are sorted by decreasing importance, and only those with a significance ≤ 0.05 are shown. The most important predictor was the D-dimer, which was the most significant among the laboratory tests, and the demographic variables used to define the prediction model. Moreover, we also found important outcome predictors among some textural features belonging to the GLOBAL, GLCM, GLSZM, GLRLM, and NGDTM families. Among the comorbidities, cardiovascular disease appears to have a significant impact on survival; it is the most significant predictor of mortality.

**Figure 2:**
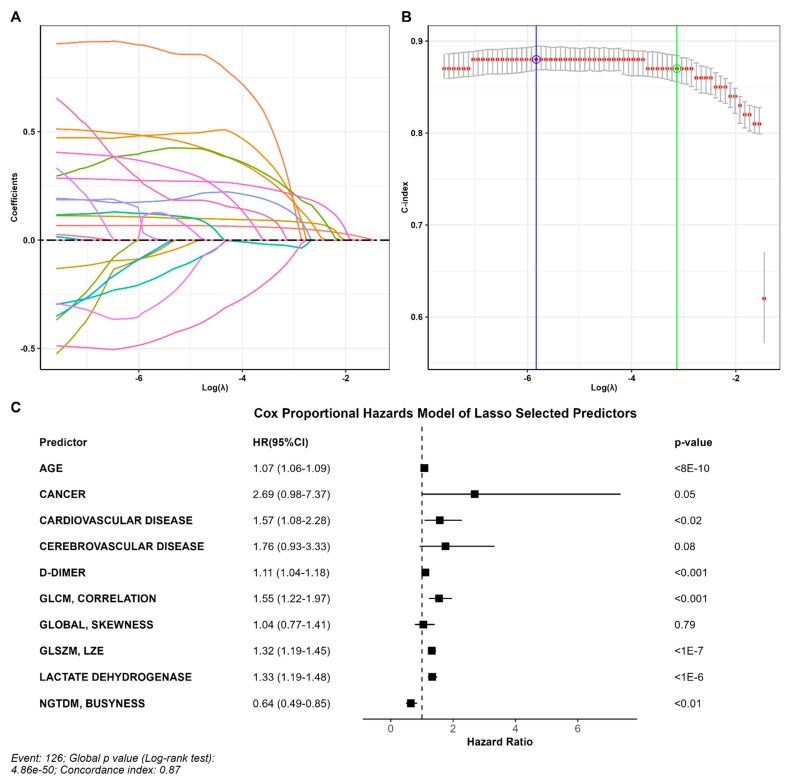
Predictors of outcome. (**A**) Coefficient profile plotted versus the log (λ). Each colored line represents the coefficient of each feature. (**B**) The C-index was plotted versus log (λ). The green circle and line locate the Lambda with minimum cross-validation error. The blue circle and line locate the point with minimum cross-validation error plus one standard deviation. (**C**) Variables that survived the LASSO regression, including age, D-dimer, LDH, three comorbidities, and four radiomic variables.

Ten out of fifty-seven variables with non-zero coefficients survived the LASSO regression and were thus included in the predictive model. The parameter producing a C-index within one standard error was 0.044, corresponding to a C-index of 0.87 (standard error = 0.014) (Figure 2A,B). Selected variables included age, D-dimer, LDH, three groups of comorbidities and four radiomic variables (Figure 2C).

Validation on the training dataset showed high agreement between the predicted and observed survival curves (Figure S2 in Supplemental Material). The unadjusted and bias-adjusted curves were similar and aligned with the dashed curve that represents the best possible relationship between the observed and predicted outcomes as estimated by the mean absolute error (MAE) of 0.01 (Figure S2, left panel; see Supplemental Material). The C-index and Brier scores were 0.872 and 0.0708, respectively. On the test dataset, the C-index, Brier score, and MAE estimated between the predicted and observed curves were 0.885, 0.056, and 0.03, respectively (Figure S2, right panel; see Supplemental Material).

**Figure 3:**
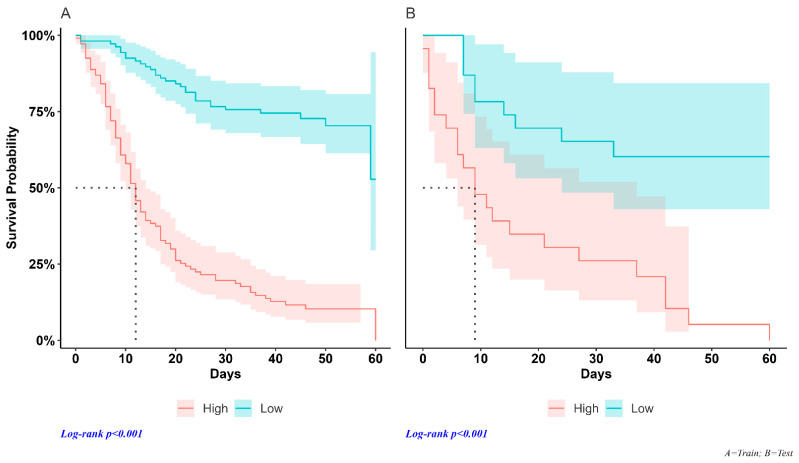
Survival curves. Training data set (**A**): the survival time of SARS-CoV-2 patients in the high-risk group differed significantly from that of the low-risk subjects, with a median of 12 days (95% CI; 10–14). The low-risk group did not achieve the 50% survival rate. Test dataset (**B**): the median survival duration of the high-risk group was 9 days (95% CI; 6–37) and low-risk patients did not reach the median survival of 50%.

We used the median individual risk score assessed using Cox regression as a cut-off point to classify patients into high- and low-risk groups in each dataset. In the training sample, median survival times were 12 days (95% CI; 10–14) and were not reached (Figure 3A) in high-risk and low-risk patients, respectively (HR = 6.59, 95% CI = 4.34–10.0, *p* < 0.001). Median survival times in the test set were 9 days (95% CI; 6–37) and were not reached (Figure 3B) in high-risk and low-risk patients, respectively (HR = 4.23, 95% CI = 1.93–9.26, *p* < 0.001).

Table 3 shows the comparison between the observed outcome and the estimated risk in the training and testing datasets. The prediction model on the testing dataset identifies 90% of true positives among subjects at risk of death, while 66% of true negatives were classified as having a low risk of an event. The risk of mortality was found to be significantly higher (Odds ratio = 13 [95% CI; 4–42], *p* < 0.0001) among the high-risk group than in the low-risk group.

## 4. Discussion

Prediction of disease severity and progression in SARS-CoV-2 patients is relevant because early intervention is significantly associated with reduced mortality [38,39]. In this study, we developed and validated a risk-scoring model based on demographics, laboratory tests, and radiomic features to predict the disease progression and survival of hospitalized patients with SARS-CoV-2. 

We implemented the model with 10 out of 57 variables selected using LASSO Cox regression and the C-index metric. The proposed model is highly predictive, identifying 90% of deceased patients in the testing set as high-risk and 66% of surviving patients as low-risk. In addition, the estimated C-index of 0.885 summarizes how well the model-predicted risk describes the observed sequence of events. The risk-estimation model included age, laboratory tests (D-dimer and LDH), and four radiomic features. 

To date, numerous studies have been conducted on CT-based radiomics features aimed at building diagnostic models to detect SARS-CoV-2 infection or predict hospital stay and outcome.

Shiri et al. demonstrated that a mixed model including CT-based radiomic features and clinical data could be used to predict survival in SARS-CoV-2 patients. Among the radiomics variables, HGLZE from GLSZM and RLNU from GLRMN showed the highest diagnostic performance (AUC = 0.73) [20]. Yang et al. reported a high diagnostic performance of radiomic variables in the classification of SARS-CoV-2 patients from other pneumonias with AUC values of 91.5%, 90.0%, 89.0%, and 87.6% for the GLCM, GLRLM, NGLDM, and GLZLM classes, respectively [40].

Radiomics variables significantly associated with outcome are able to describe the distribution of voxel intensities within the image included in a mask that defines the region of interest. The most significant predictors are those sensitive to changes in the distribution of voxel intensity associated with disease progression. In SARS-CoV-2 pneumonia, the extent of ground glass opacity is generally greater in patients who progress to more severe stages of the disease. In these cases, the voxel-intensity distribution is more uniform and leads to a different estimate of the texture parameters compared to less-involved patients.

The variables used to estimate the risk of developing critical illness due to SARS-CoV-2 infection are generally available in the early stages of hospitalization. Risk estimation in this phase could support clinicians in planning a treatment strategy by allowing them, in higher-risk cases, to allocate resources for more aggressive treatments or admit patients to intensive care units; equally, it would enable physicians to adopt a “watch and wait” approach to low-risk cases. 

Previous studies have reported the impact of age on SARS-CoV-2 mortality. A meta-analysis demonstrated age’s decisive effect on mortality [9]. A 60% higher risk of mortality was reported in subjects aged >80 years [9]. As expected, the age of non-surviving patients in our study was significantly higher than that of survivors (median 80 years, 95% CI: 51–107 vs. 59 years, 95% CI: 20–94; *p* < 0.001). Age was an important predictor of disease outcome (Figure 1) and survived the LASSO regression. It thus contributed to the implemented prediction model.

D-dimer was associated with poor outcomes for patients with SARS-CoV-2, presumably due to the increased likelihood of their developing pulmonary embolisms when they had D-dimer levels above 2590 ng/mL [41]. According to recently published papers [42,43], reinforced by the results for our sample, D-dimer was the variable most strongly associated with patient outcome (Figure S1) as suggested by measured levels of 6.006 vs. 1.133 ng/mL for deceased patients and survivors, respectively.

Similarly, elevated lactate dehydrogenase (LDH) levels have been associated with worse outcomes in patients with viral infections [44,45]. Deceased patients in our study had significantly higher LDH levels than survivors (Table 1), and it was selected using LASSO regression for the survival-prediction model.

Reports in the literature have documented that chronic comorbidities are associated with an increased risk of poor prognosis and a fatal outcome associated with SARS-CoV-2 [10]. Similarly, in our model, pre-existing comorbidities (including cardiovascular and cerebrovascular diseases, cancer, haematological diseases, and chronic obstructive pulmonary disease) were significant predictors of severity of disease and death following SARS-CoV-2 infection. Among the comorbidities, cardiovascular disease was the strongest predictor of mortality in our study sample, with a 4.42-fold higher risk of poor prognosis, in line with the findings of several meta-analyses [46,47,48,49]. 

CT is the most widespread imaging modality to play a key role in the diagnosis and assessment of the prognosis of patients with SARS-CoV-2 [50]. However, CT findings (such as ground-glass opacities or consolidation) are not specific to SARS-CoV-2, as these can also be found in other diseases associated with a lower risk of death, such as seasonal influenza.

Innovative methods of quantitative image analysis (such as radiomics) can provide an operator-independent semi-quantitative approach by describing spatial and temporal information derived from images (CT, MRI, and PET/CT). Until now, radiomics have been applied in medical fields such as oncology and to specific disorders such as neurodegenerative disease [51,52,53]. Lately, it has also been used to support “digital biopsy”, a non-invasive tissue-characterization technique.

Previous studies have reported the potential use of CT radiomic features to better characterize pulmonary involvement in patients with SARS-CoV-2. Spatial information measured with radiomic features can be used to support differential diagnosis between COVID and non-COVID disease [54], as well as for modelling risk of death and predicting survival [24].

In our study, we selected four radiomic features (Global_Skewness, GLCM_Correlation, GLSZM_LZE, NGTDM_Busyness) to model a risk profile with significant discriminative capabilities for patient outcome. Indeed, selected variables were significantly associated with patient outcome in multivariate logistic regression (*p* < 0.001). These features contribute to risk modelling by providing quantitative information on lung CT-signal intensity and heterogeneity in SARS-CoV-2 patients. 

A systematic review of existing prognostic models identified several were designed to support diagnosis and predict mortality among patients hospitalized for SARS-CoV-2 [7]. Most of the studies reported that predictive models implemented with CT images and/or clinical variables were combined differently depending on the available data. 

Only a few studies combined radiomics, demographics, comorbidities, and laboratory tests as potential predictor candidates. The main disadvantage of these studies is their small sample size, which exposes the results to a high risk of bias due to inappropriate evaluation of the predictive performance of the test dataset.

Our study included 694 patients with complete radiomic and clinical datasets. The predictors needed to calculate the risk of developing serious disease are usually available within the first few hours of hospital admission. Using these variables, the model can estimate the risk of mortality, identifying 90% of non-survivors in the study sample. The availability of this information could be useful for optimising treatment planning according to the estimated risk when patients are admitted to hospital.

## 5. Study Limitations

The major limitation of the study lies in the lack of external validation using a dataset obtained from another hospital. Although our validation was performed on a test set not used for training, to build a robust model and obtain reliable performance evaluation, it would be advisable to validate the model using data from different sources.

Our model is not available as a ready-to-use software package. The study was designed to define and validate a predictive risk model to be subsequently produced as a usable application in clinical practice. To this end, we used commonplace open-source statistical software. These packages facilitate the easy transfer of the method into clinical practice.

Our study lacks information on out-of-hospital mortality. Therefore, mortality may be underestimated due to the death of patients after discharge. 

Future development of the results and findings of this study could include i) validating the predictive model on an external dataset and ii) re-evaluating the time-to-event analysis if new data become available. Furthermore, considering the availability of different machine learning algorithms used in different clinical settings including the SARS-CoV-2 pandemic, a useful future aim will be to compare the performance of predictive models based on different approaches.

## 6. Conclusions

We developed a predictive model of mortality in a sample of 694 SARS-CoV-2 patients using demographic, CT-radiomic, and laboratory tests. We calibrated and validated the model by randomly splitting the sample into training and test datasets. We implemented the final model with a combination of 10 variables including age, D-dimer, LDH, preexisting comorbidities such as cancer and cardiovascular and cerebrovascular diseases, and four radiomic features. The model was able to correctly identify 90% of non-survivors. Identifying high-risk individuals with predictors usually available within the first few hours of hospital admission could be useful in cases of widespread disease to enable more effective allocation of available resources.

## Figures and Tables

**Table 1:** Clinical characteristics of SARS-CoV-2 population in living and deceased subjects.

	Observed Outcome				
Variable	Overall N = 694	Alive N = 537 (77%)	Deceased N = 157 (23%)	Statistic	*p*-Value ^1^
Age				213	<0.001
Median (Range)	64 (20–107)	59 (20–94)	80 (51–107)		
Gender, n (%)				0.07	0.8
Female	247 (36%)	193 (36%)	54 (34%)		
Male	447 (64%)	344 (64%)	103 (66%)		
Hospital stay				10	0.001
Median (Range)	11 (3–86)	10 (3–86)	13 (3–62)		
CT findings, n (%)				11	0.001
Negative/Atypical	284 (41%)	238 (44%)	46 (29%)		
Typical	410 (59%)	299 (56%)	111 (71%)		
C-reactive protein				19	<0.001
Median (Range)	3 (0–37)	3 (0–27)	5 (1–37)		
Lactate dehydrogenase				97	<0.001
Median (Range)	486 (78–3745)	484 (78–3745)	494 (141–3631)		
D-dimer				68	<0.001
Median (Range)	1133 (96–152,015)	1133 (96–55,660)	6006 (168–152,015)		

^1^ One-way ANOVA; Pearson’s chi-squared test.

**Table 2:** Diseases associated with a high risk of mortality in SARS-CoV-2 infection.

Comorbidity	Alive, N = 537 ^1^	Deceased, N = 157 ^1^	Odds Ratio ^2^	95% CI ^2,3^	*p*-Value ^2^
Cardiovascular disease	90 (17%)	74 (47%)	4.42	2.95, 6.63	<0.001
Cancer	2 (0%)	9 (6%)	16.2	3.30, 155	<0.001
Cerebrovascular disease	9 (2%)	14 (9%)	5.72	2.25, 15.3	<0.001
Haematological disease	14 (3%)	13 (8%)	3.36	1.42, 7.91	0.003
Chronic obstructive pulmonary disease	24 (4%)	16 (10%)	2.42	1.17, 4.90	0.011
Blood cancer	11 (2%)	1 (1%)	0.31	0.01, 2.14	0.3
Hypertension	66 (12%)	16 (10%)	0.81	0.42, 1.47	0.6
Type 2 diabetes	77 (14%)	20 (13%)	0.87	0.49, 1.50	0.7
Obesity	11 (2%)	3 (2%)	0.93	0.16, 3.59	>0.9

^1^ n (%); ^2^ Fisher’s Exact Test for Count Data; and ^3^ CI = Confidence Interval.

**Table 3:** Bivariate analysis of model performance by dataset.

		Observed								
Predicted	N	Deceased ^1^	Alive ^1^	X^2^	p ^2^	SS 95%CI ^3^	SP 95%CI ^4^	PPV 95%CI ^5^	NPV 95%CI ^6^	OR 95%CI ^7^
Training	556			140	2.1 × 10^−32^	97 (92, 99)	64 (59, 68)	44 (38, 50)	99 (96, 100)	48 (18, 125)
High risk		122 (97%)	156 (36%)							
Low risk		4 (3.2%)	274 (64%)							
Test	138			24	9.8 × 10^−7^	90 (74, 98)	62 (52, 71)	41 (29, 53)	96 (88, 99)	13 (4, 42)
High risk		28 (90%)	41 (38%)							
Low risk		3 (9.7%)	66 (62%)							

^1^ n (%), ^2^ Pearson’s chi-squared test, ^3^ sensitivity, confidence interval, ^4^ specificity, confidence interval, ^5^ positive predictive value, confidence interval, ^6^ negative predictive value, confidence interval, ^7^ odds ratio, and confidence interval.

## Data Availability

Data not available due to privacy issues.

## Supplementary Materials

The following supporting information can be downloaded at https://www.mdpi.com/article/10.3390/jcm12227164/s1, Figure S1: Predictors impact ranking on mortality. Higher Chi-squared estimstes are suggestive of a greater association with clinical outcome. Label on Y axis show predictor name and P value; Figure S2: Calibration curves from regression model predictions by datdaset.
