# Supplementary material for: Multivariable Risk Modelling and Survival Analysis with Machine Learning in SARS-CoV-2 Infection
Source: J Clin Med. 2023 Nov 18;12(22):7164. doi: 10.3390/jcm12227164 (PMC10672177; doi:10.3390/jcm12227164)
Supplement: Supplementary file 1 [file jcm-12-07164-s001.zip › jcm-2682594-supplementary.pdf]

Supplemental Matherial

- Figure S1
- Figure S2
- Figure Legends

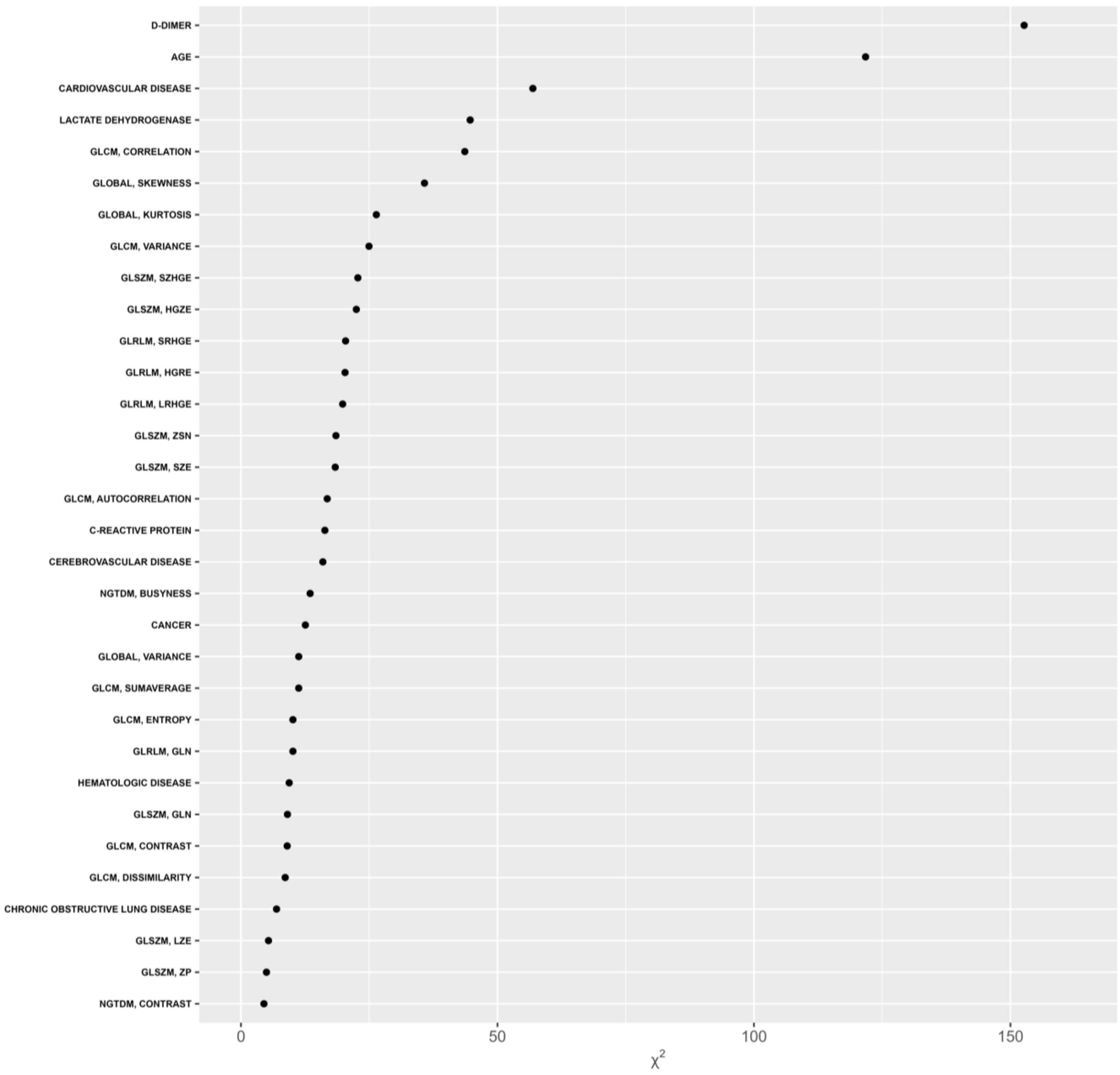

Figure S1. Predictors impact ranking on mortality. Higher Chi-squared estimstes are suggestive of a greater association with clinical outcome. Label on Y axis show predictor name and P value.

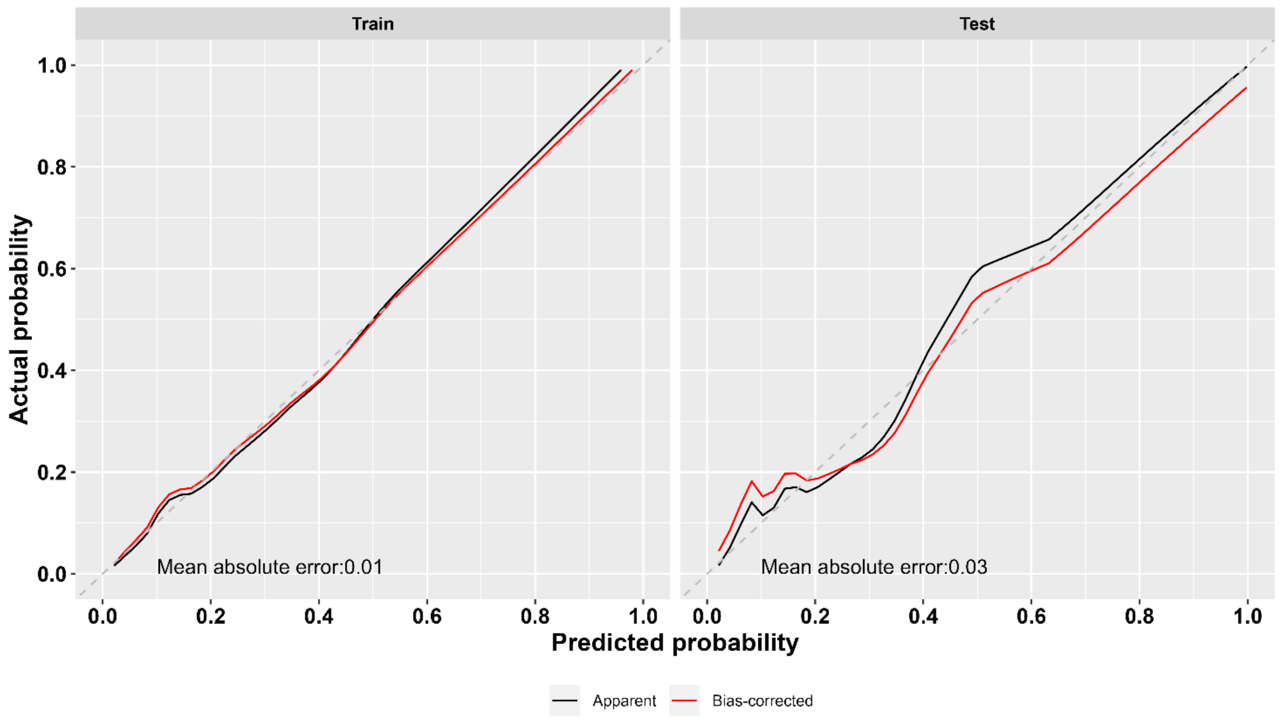

Figure S2. Calibration curves from regression model predictions by datdaset.

Figure Legends

Figure S1. Predictors importance. Relevance of predictors based on the Wald test for multivariable logistic regression. Only those with a significance ≤ 0.05 are shown, in order of importance. The D-dimer was the most important predictor. Relevant predictors were several textural features belonging to the GLOBAL, GLCM, GLSZM, GLRLM and NGDTM families. Among comorbidities, cardiovascular disease was the strongest risk factor.

Figure S2. Calibration curves. Training validation (left panel) shows high agreement between the predicted and observed survival curves. The unadjusted and bias-adjusted curves were similar to the dashed curve representing the best possible relationship between observed and predicted outcome as estimated by the mean absolute error (MAE) of 0.01. In the test set validation (right panel), a sample of 138 SARS-CoV-2 patients randomly assigned, the mean MAE was 0.03.
